# Do Patients Treated for Colorectal Cancer Benefit from General Practitioner Support? A Video Vignette Study

**DOI:** 10.2196/jmir.4942

**Published:** 2015-11-05

**Authors:** Irene Ngune, Moyez Jiwa, Alexandra McManus, Richard Parsons, Georgina Pagey, Rupert Hodder

**Affiliations:** ^1^ Faculty of Health, Engineering and Science Edith Cowan University Perth Australia; ^2^ School of Medicine University of Notre Dame NSW Australia; ^3^ Faculty of Health Sciences Curtin University Perth Australia; ^4^ Curtin University Perth Australia; ^5^ Department of Surgery Sir Charles Gardiner Hospital Perth Australia

**Keywords:** colorectal cancer, general practice, Internet survey, side effects, video vignettes

## Abstract

**Background:**

Patients who have been treated for colorectal cancer in Australia can consult their general practitioner (GP) for advice about symptoms or side effects at any time following their treatment. However, there is no evidence that such patients are consistently advised by GPs, and patients experience substantial unmet need for reassurance and advice.

**Objective:**

To explore the patient management options selected by GPs to treat a set of patients describing their symptoms following treatment for colorectal cancer.

**Methods:**

This was an Internet-based survey. Participants (GPs) viewed 6 video vignettes of actors representing patients who had been treated for colorectal cancer. The actor-patients presented problems that resulted from their treatment. Participants indicated their diagnosis and stated if they would prescribe, refer, or order tests, based on that diagnosis. These responses were then rated against the management decisions for those vignettes as recommended by a team of colorectal cancer experts.

**Results:**

In total, 52 GPs consented to take part in the study, and 40 (77%) completed the study. Most GPs made a diagnosis of colorectal cancer treatment side effects/symptoms of recurrence that was consistent with the experts’ opinions. However, correct diagnosis was dependent on the type of case viewed. Compared with radiation proctitis, GPs were more likely to recognize peripheral neuropathy (odds ratio, OR, 4.43, 95% CI 1.41-13.96, *P*=.011) and erectile dysfunction (OR 9.70, 95% CI 2.48-38.03, *P*=.001), but less likely to identify chemotherapy-induced fatigue (OR 0.19, 95% CI 0.08-0.44). GPs who had more hours of direct patient care (OR 0.38, 95% CI 0.17-0.84, *P*=.02), were experienced (OR 9.78, 95% CI 1.18-8.84, *P*=.02), and consulted more patients per week (OR 2.48, 95% CI 1.16-5.30, *P*=.02) suggested a management plan that was consistent with the expert opinion.

**Conclusions:**

In this pilot study, years of experience and direct patient contact hours had a significant and positive impact on the management of patients. This study also showed promising results indicating that management of the common side effects of colorectal cancer treatment can be delegated to general practice. Such an intervention could support the application of shared models of care. However, a larger study, including the management of side effects in real patients, needs to be conducted before this can be safely recommended.

## Introduction

Colorectal cancer is the second most commonly diagnosed adult cancer in Australia [1]. One in 12 people in Australia will develop colorectal cancer in their lifetime [2]. Most people with colorectal cancer survive more than 5 years and die of unrelated causes [3]. The treatment of colorectal cancer may include surgery, radiotherapy, and chemotherapy. In the months and years following treatment, people may experience a number of troublesome side effects or symptoms and signs related to cancer recurrence. Many patients may experience bowel dysfunction, sexual dysfunction, urinary dysfunction, and fatigue, among other difficulties [4].

Post-treatment follow-up is provided in secondary settings in some instances; however, this follow-up may only be for a short period for some patients, after which they are encouraged to see their general practitioner (GP) about any ongoing problems [5]. Previous studies have demonstrated that cancer patients consult a GP routinely in the months and years after treatment for colorectal cancer, even those with scheduled follow-up visits at the hospital [6]. Colorectal cancer patients may contact their GP for a range of symptoms, such as radiation proctitis, urinary incontinence/urgency, fatigue, erectile dysfunction, and symptoms of recurrence [7]. To address the needs of patients treated for colorectal cancer, the GP needs to be knowledgeable about the recommended treatment for side effects of colorectal cancer treatment and the signs and symptoms that merit referral for further specialist treatment.

In this pilot video vignette study, we aim to explore the impact of a variety of clinical and respondent characteristics of GPs’ decisions to treat colorectal cancer patients experiencing treatment side effects or symptoms of recurrence of their cancer.

## Methods

### Participants

Ethical approval was obtained from the Curtin Human Research Ethics Committee (HR 42/2012). Participants were then recruited from a network of 100 GPs across Australia. GPs were emailed invitations and the initial emails were supplemented with follow-up personal invitations to the invitees who did not initially respond. Participants were remunerated with AUD $50 for their contribution.

### Video Vignettes

Six video vignettes were developed, each presenting a potential side effect related to treatment for colorectal cancer or features of cancer recurrence (see Multimedia Appendix 1 for an example). The range of scenarios was based on the most common side effects reported by colorectal cancer patients (see Multimedia Appendix 2). The identification and validation of these side effects were reported in a different phase of this project [8]. Each vignette depicted a patient with clear indications for specific management, including referral, prescription, reassurance, and/or investigation. The vignettes were developed by 4 GPs, a radiation therapist, a medical oncologist, and a surgeon. This expert panel also suggested the management of each case, with details of prescription, referral for specialist treatment, and laboratory investigations (see Multimedia Appendix 3).

The vignettes were then prepared as a short video monologue by an actor-patient. The video included an off-camera commentary by an actor-doctor describing relevant signs to be found on clinical examination. Participation in the study was via the Internet. Information on the actor-patient’s medical history, family history, medication history, and physical assessment was offered at the onset of each video. Participants were asked the following 4 questions after watching each video vignette:

1. “What is your diagnosis?”

2. “Would you prescribe something? If so, what would you prescribe?”

3. “Would you refer the patient? If so, to whom?,” and

4. “Would you order tests? If so, which tests?”

The participants’ responses were then assessed by a team of 2 researchers (IN and GP) against the experts’ opinion. Where differences arose, the third researcher (MJ) validated the assessment.

### Sample Size and Statistical Analysis

The main aim of this study was to evaluate the treatment GPs offer to standardized patients presenting with side effects of colorectal cancer treatment or symptoms of recurrence. Each GP reviewed the same set of 6 video vignettes and responded to the 4 aforementioned binary (Yes/No response) questions regarding prescription of medication, referral for further treatment, or ordering of tests. Each of these 4 questions was analyzed in a separate general estimating equation (GEE) model, with the binary response as the dependent variable, and the subject named as the “random effect.” The GEE model is appropriate to this design as it takes into account the correlation between responses from the same GP across the 6 vignettes.

The estimated sample size required to give adequate power to detect associations with the independent variables is difficult to estimate, but depends on the expected response proportions (proportions of positive responses) and the correlations between responses belonging to the same respondent. In the absence of pilot data on which these quantities might have been estimated, a sample of 40 GPs was sought (who would provide 240 observations in total). This projected number cannot be mathematically justified in the absence of pilot data. However, in a standard regression model, a sample of 120 uncorrelated measurements should be adequate to identify an independent variable exhibiting a moderate effect size with 80% power [9]. It was assumed that doubling the number of observations would be adequate to compensate for the internal correlations in the dataset.

Each of the GEE models initially included the following independent variables: age, years of GP experience, recognized specialty qualification with the Royal Australian College of General Practitioners (RACGP) (Fellowship of the RACGP or FRACGP), number of patients consultations per week and patient consultations hours per week. A backward elimination method was used to arrive at the final model. This method involved dropping the least variable, one at a time, until all variables remaining in the model were ly associated with the outcome.

SPSS Version 21 software was used to perform the analysis. Following convention, a *P* value less than .05 was taken to indicate a statistically association in all tests.

## Results

### Demographics

In total, 52 GPs participated in the project, but only responses of participants who completed the entire survey (40 GPs) were considered for analysis of the primary outcomes. Those who participated in the study were younger than Australian GPs generally (mean age 36.9 years vs 50.5 years), and a greater proportion were females (57.7%, 30/52, vs 39.1%). The demographic details of the respondents are shown in Table 1.

### Diagnosis Consistent With Expert Opinion

The colorectal cancer video vignettes were presented 240 times in the study (40 GPs × 6 vignettes). Of the 240 diagnoses made by the GPs, 168/240, 70.0% (range 35-95%), were consistent with the expert diagnosis. This consistency was observed more for erectile dysfunction (38/40, 95%), peripheral neuropathy (36/40, 90%), and tumor recurrence (31/40, 78%), compared with urinary dysfunction (23/40, 58%) and cancer-related fatigue (14/40, 35%). A higher proportion of correct diagnoses were made by GPs who worked more than 60 patient-care hours per week (15/18, 83%), those who held a GP fellowship (101/138, 73.2%), and those who had less than 10 years of experience (1-2 years 71/96, 74%; 3-10 years 53/72, 74%).

A multivariate GEE analysis was carried out to determine whether a correct diagnosis depended on the case itself or characteristics of the GP. There were some statistically differences in the diagnosis of the cases. Compared with radiation proctitis, GPs were more likely to identify cases with chemotherapy-induced peripheral neuropathy (odds ratio, OR, 4.43, 95% CI 1.41-13.96, *P*=.01) or erectile dysfunction (OR 9.70, 95% CI 2.48-38.03, *P*=.001), but were less likely to recognize chemotherapy-induced fatigue (OR 0.19, 95% CI 0.08-0.44, *P*=.001). In addition, younger GPs (<30 years of age; OR 2.64, 95% CI 1.12-6.22, *P*=.03) and those who held a GP fellowship (OR 3.26, 95% CI 1.62-6.62, **P*<.*001) were more likely to identify cases consistent with the expert opinion. The demographic characteristics of the GP did not have any influence on their ability to recognize colorectal cancer treatment side effects or symptoms of recurrence. Details of the factors associated with correct diagnosis are displayed in Table 2.

**Table 1:** Participant demographic information (N=52).

Characteristics			Study sample	National population^a^ Mean/%
**Demographics**				
	Age (years), mean (SD)		36.9 (10.5)	50.5
	Years of GP experience, mean (SD)		7.0 (9.7)	
	**Gender, n (%)**			
		Male	22 (42.3)	60.9
		Female	30 (57.7)	39.1
	Registrars (GPs in training), n (%)		17 (32.7)	3.8
	FRACGP, n (%)		28 (53.8)	56.8
**Practice demographics**				
	Practice accredited, n (%)		52(100.0)	88.6
	**Clinic remoteness, n (%)**			
		Major city	36 (69.2)	71.1
		Nonmajor city	16 (30.8)	28.9
	**Clinic location, n (%)** ^b^			
		Capital	27 (51.9)	
		Other metropolitan	14 (26.9)	
		Large rural	6 (11.5)	
		Small rural	4 (7.7)	
		Remote center	1 (1.9)	
**GP position in the practice, n (%)**				
		Principal	8 (15.4)	
		Nonprincipal	35 (67.3)	
		Others	9 (17.3)	
**Patient consultations**				
	**Patient consultations per week, n (%)**			
		<100	22 (42.3)	
		100-149	21 (40.4)	
		≥150	9 (17.3)	
	**Patient consultations hours per week, n (%)**			
		<11	10 (19.2)	1.2
		11-20	4 (7.7)	12.2
		21-40	24 (46.2)	53
		41-60	14 (26.9)	33.5
	**Non-English consultations, n (%)**			
		No	45 (86.5)	
		Yes, <25%	7 (13.5)	24.5

^a^Sourced from national data when available [10].

^b^Classification based on Rural, Remote and Metropolitan Area classification [11,12].

**Table 2:** Factors associated with correct diagnosis (outcome consistent with expert opinion).^a^

Variable		n/N (%)	Odds ratio	95% CI	*P*
**Age**					
	31 years or older	103/156 (66.0)	1 (reference)		
	30 years or younger	67/84 (79.8)	2.64	1.12-6.22	.0262
**Years of practice**					
	1-5	101/132 (76.5)	1 (reference)		
	5 or more	69/108 (63.9)	0.42	0.20-0.87	.0189
**GP holds a fellowship**					
	No	69/102 (67.6)	1 (reference)		
	Yes	101/138 (73.2)	3.26	1.62-6.54	.0009
**Case vignette**					<.0001^b^
	1. Peripheral neuropathy	36/40 (90.0)	4.43	1.41-13.96	.0110
	2. Erectile dysfunction	38/40 (95.0)	9.70	2.48-38.03	.0011
	3. Urinary dysfunction	23/40 (57.5)	0.54	0.20-1.46	.2227
	4. Tumor recurrence	31/40 (77.5)	1.55	0.48-5.06	.4663
	5. Cancer-related fatigue	14/40 (35.0)	0.19	0.08-0.44	.0001
	6. Radiation proctitis	28/40 (70.0)	1 (reference)		

^a^The independent variable was a correct response. For example, in the first analysis, respondents who were aged 30 or younger were ly more likely (OR 2.64) to give a correct diagnosis than the older participants. The numbers in the third column show the number and percentage of correct responses within the group defined by the row. For example, 80% (67/84) of the diagnoses from people aged 30 or under were correct compared with 66% (103/156) for the older group.

^b^ *P* value for the variable as a whole.

### Management Consistent With Expert Opinion

Management of the cases according to the expert opinion was categorized into 3 domains, namely, (1) refer, (2) prescribe, and (3) order tests.

#### Refer

Only 5/6 cases were deemed by the experts to require referral. The analysis of this variable used only the records relating to these vignettes (n=200 observations), as it was far more important that the GP should refer when a referral was considered important than they should do so when it was considered unimportant. Of these 200 observations, only 86/200, 43% (range 18-60%), were consistent with the expert opinion (so 57%, 114/200, did not refer, when it was considered appropriate to do so). This inconsistency occurred more frequently for the cases of erectile dysfunction, radiation proctitis, and peripheral neuropathy, with only 18% (7/40), 38% (15/40), and 43% (17/40) of these cases correctly referred, respectively.

Similarly, only 38% (15/40) of the referrals made by GPs who worked more than 60 patient care hours per week and 33% (26/80) of those made by GPs who had 1-2 years of experience were consistent with expert opinion. The results of the regression analysis revealed that the number of patient contact hours done by a GP per week and years of practice influenced GPs’ decisions to refer. Compared with GPs who worked up to 40 hours, GPs who worked more than 40 hours per week were more likely (OR 0.38, 95% CI 0.17-0.84, *P*=.02) to refer the patient, in agreement with the expert opinion. GPs with 1 year of experience (OR 0.30, 95% CI 0.13-0.66, *P*=.003) were less likely to refer according to expert opinion. Details of the factors associated with correct referrals are displayed in Table 3.

#### Prescribe

Of the 120 observations made by the GPs to correctly prescribe, only 39% (n=47; range 27-70%) of the prescriptions were consistent with the expert opinion. The only cases with higher proportion of GPs who gave prescriptions that were consistent with expert opinion were erectile dysfunction (28/40, 70%).

The results of the regression analysis show that, compared with radiation proctitis, GPs were more likely to offer a prescription for erectile dysfunction that was consistent with expert opinion (OR 1.27, 95% CI 0.47-3.42, *P*=.63). However, this association was not statistically .

Details of the factors associated with correct prescription are displayed in Table 3.

#### Order Tests

Of the 160 observations made by the GPs to order tests, at least 50% (80/160) were consistent with the expert opinion (average 36, range 10-85%). This consistency was observed more for chemotherapy-induced fatigue (33/40, 83%) and tumor recurrence (32/40, 80%) compared with radiation proctitis (4/40, 10%) and urinary dysfunction (16/40, 40%). Sixty-four percent (23/36) of tests ordered by GPs who had more than 150 patient consultations per week (OR 2.48, 95% CI 1.16-5.30, *P*=.02) were consistent with the expert opinion.

Regression analysis results showed that compared with ordering tests for radiation proctitis, GPs were more likely to order tests for urinary dysfunction (OR 6.33, 95% CI 1.58-25.42, *P*=.01), tumor recurrence (OR 40.02, 95% CI 10.29-155.68, *P*<.001), and chemotherapy-induced fatigue (OR 47.29, 95% CI 11.47-195.00, *P*<.001). Details of the factors associated with correct ordering of tests by GPs are displayed in Table 3.

**Table 3:** Factors associated with management that is consistent with expert opinion.^a^

Outcome	Variable		n/N (%)	Odds ratio	95% CI	*P* value
**Prescribe**	**FRACGP**					
		No	33/51 (64.7)	1 (reference)		
		Yes	32/69 (46.4)	0.41	0.17-1.00	.0508
	**Case vignette**					<.0001^b^
		1. Peripheral neuropathy	11/40 (27.5)	0.19	0.08-0.47	.0003
		2. Erectile dysfunction	28/40 (70.0)	1.27	0.47-3.42	.6388
		6. Radiation proctitis	26/40 (65.0)	1 (reference)		
**Refer**	**Years of practice**					
		1	15/55 (27.3)	0.30	0.13-0.66	.0027
		2 or more	71/145 (49.0)	1 (reference)		
	**Hours of patient contact per week**					
		Up to 40	69/145 (47.6)	1 (reference)		
		41 or more	17/55 (30.9)	0.38	0.17-0.84	.0165
	**Case vignette**					.0005^b^
		1. Peripheral neuropathy	17/40 (42.5)	1.26	0.58-2.71	.5632
		2. Erectile dysfunction	7/40 (17.5)	0.33	0.10-1.04	.0582
		3. Urinary dysfunction	23/40 (57.5)	2.44	0.94-6.35	.0680
		4. Tumour recurrence	24/40 (60.0)	2.73	0.98-7.60	.0542
		6. Radiation proctitis	15/40 (37.5)	1 (reference)		
**Order tests**	**Number of patients seen per week**					
		Less than 150	62/124 (50.0)	1 (reference)		
		150 or more	23/36 (63.9)	2.48	1.16-5.30	.0191
	**Case vignette**					<.0001^b^
		3. Urinary dysfunction	16/40 (40.0)	6.33	1.58-25.42	.0092
		4. Tumour recurrence	32/40 (80.0)	40.02	10.29-155.68	<.0001
		5. Cancer-related fatigue	33/40 (82.5)	47.29	11.47-195.00	<.0001
		6. Radiation proctitis	4/40 (10.0)	1 (reference)		

^a^The table shows the results of 3 GEE models. For each analysis, the dependent variable was a correct response. The numbers in the third column show the number and percentage of correct responses within the group defined by the row.

^b^ *P* value for the variable as a whole.

## Discussion

### Preliminary Findings

In this study, we have explored the impact of a variety of clinical and respondent characteristics on GPs’ decisions to treat patients with treatment side effects or symptoms of recurrence of colorectal cancer. Peripheral neuropathy, fatigue, bowel dysfunction, urinary dysfunction, tumor recurrence, and sexual dysfunction are common presentations of patients with colorectal cancer in general practice [13]. Our data indicate that GPs correctly diagnosed most of these conditions, with the exception of chemotherapy-induced fatigue. This would be expected, as in most cases, fatigue presents as a manifestation of other underlying conditions and it is also difficult to diagnose [14]. Although participating GPs did not recognize fatigue, the regression results showed that they ordered tests to explore underlying conditions that were consistent with the expert suggestions. Our results also indicate that younger GPs (<30 years of age) and those who held a GP fellowship were more likely to identify cases consistent with the expert opinion. The recency of training may have contributed to their level of awareness of colorectal cancer treatment-related problems. However, given the modest sample size we are cautious about drawing firm conclusions on this point.

Suggestions for management plans for these conditions were, however, not consistent with expert opinion in all the applicable categories of management (refer, prescribe, and order tests) for the specific cases. From the regression analysis, we were able to conclude that compared with radiation proctitis, tumor recurrence, fatigue, and urinary dysfunction were more likely to be managed according to the experts’ opinions. There were marked deviations from the experts’ suggestions for the cases of erectile dysfunction and peripheral neuropathy. For example, for erectile dysfunction, practitioners were less likely to refer back to the specialist, but offered appropriate medication. Similarly, there were deviations from expert management for peripheral neuropathy and urinary dysfunction. Such deviations from expert opinion have been reported previously in similar studies with prostate cancer patients [15].

The differences in management between the participants and the expert panel were less marked for the management of tumor recurrence. This may be expected, as most patients first present to a GP before the cancer diagnosis [16] or with symptoms of recurrence even with ongoing management by their specialist [6]. It is therefore plausible that the GPs were well experienced in recognizing and making appropriate decisions related to tumor recurrence.

Regression analysis also suggested that there were other influential variables that had an impact on the management of these conditions. These were some of the demographic characteristics of the participants; in particular, the number of patient contact hours and years of experience. GPs with less than 1 year of experience were less likely to manage patients according to expert opinion. This was expected for patients treated for colorectal cancer, because many of these problems are likely to present infrequently when patients are still being followed by their specialist. Few doctors will have encountered them previously unless they work full-time and/or have been practicing for a longer period.

A number of approaches have been reported in the literature to promote consistent and reliable management of chronic conditions in primary care [6,15,17]. A few of these have focused specifically on the knowledge of GPs [15], and others have reported that attitudes and beliefs are important in the context of a cancer diagnosis [6]. These issues were not evaluated in this study. For example, we were unable to report on the participants’ attitude to the management of patients following treatment and whether they felt this role extended to investigating and treating conditions that may have resulted from specialist treatment.

Finally, we could not identify any practitioners who had any specialist training in colorectal cancer. However, all participants were working as GPs when they participated in this study and it is reasonable to assume that there were a negligible number with specialist training in a specific cancer.

This pilot study had a modest sample size of 240 observations, which was chosen on the basis that this number would be adequate to estimate the proportion of occasions on which at least one problem was correctly identified or managed with a reasonable precision (approximately ±10%). This was not true of all management modalities. In some cases, the number of observations was very low, as evidenced by very wide confidence limits, as shown in Table 3. Therefore, a much larger randomized study would be required to test our objectives robustly. In addition, some of the participants’ demographic characteristics differed from the national average and this may limit generalizability of the findings.

### Conclusions

In this pilot study, years of experience and direct patient contact hours had a more and positive impact on the management of patients. This study also showed promising results that management of the common side effects of cancer colorectal treatment could be delegated to general practice. Such an intervention could support the application of shared care models of care. However, a larger study, including the management of side effects in real patients, needs to be conducted before it can be safely recommended.

## Appendix

Multimedia Appendix 1 An Example of the Video Vignettes Used (.mov Movie File).

Multimedia Appendix 2 Details of Patients Presented in the Video Vignettes.

Multimedia Appendix 3 Specific Recommendations for Management of Cases.

## Abbreviations

CRC: colorectal cancer
FRACGP: Fellowship of the RACGP
GEE: general estimating equation
GP: general practitioner
OR: odds ratio
RACGP: Royal Australian College of General Practitioners
