# Supplementary material for: Do Patients Treated for Colorectal Cancer Benefit from General Practitioner Support? A Video Vignette Study
Source: J Med Internet Res. 2015 Nov 5;17(11):e249. doi: 10.2196/jmir.4942 (PMC4642383; doi:10.2196/jmir.4942)
Supplement: Multimedia Appendix 2 [file jmir_v17i11e249_app2.pdf]

Appendix 1: Details of Patients Presented in the Video Vignettes.

Details of patients presented in the video vignettes
**Case 1:** James, 58 years of age, married and works as a plumber. Has high BP but now stable. Diagnosed with bowel cancer (splenic flexure mucinous adenocarcinoma, no metastases in 21 lymph nodes, pT4b, N0, M0) 18 months ago and completed treatment 12 months ago (treatment offered: left hemicolectomy, chemotherapy—flurouracil + leucovorin + oxaliplatin FOLFOX).<br>Presents with pain and a tingling sensation in his fingertips and toes that has markedly interfered with his work. He complains of having trouble with grasping items with his fingers. On examination, there is no jaundice, anaemia, cyanosis, oedema, or lymphadenopathy. Neuro examination normal; upper limb sensation moderate on light touch; reduced from 5 cm below elbow; reflexes absent in wrist, elbow +; temp reduced from 5 cm below elbow; position sense abnormal; coordination: poor fine motor movements, unable to unbutton buttons.<br>Diagnosis: Chemotherapy-induced peripheral neuropathy
**Case 2:** David, 60 years of age, maintenance worker at a school, previous divorce and now in a new a new relationship for the last six months. Presented with erectile dysfunction that affects his relationship. He is aware such a problem would occur following surgery. His urine stream is normal. He was diagnosed with rectal cancer (mid to lower rectal mass, T3, N2, M0) and completed treatment 12 months ago (treatment offered: anterior resection, ostomy; radiotherapy—long course: 5/52). He has no family history of diabetes, random blood sugar normal, full blood count normal, and urea/electrolyte/creatinine normal; recent carcinoembryonic antigen 3 ng/ml and computerised tomography (CT) scan of abdomen normal. On examination normal, perianal reflexes intact. No jaundice, anaemia, cyanosis, oedema, or lymphadenopathy. Vital signs are within normal ranges.<br>Diagnosis: Erectile dysfunction secondary to LAR
**Case 3:** Margaret, 45 years of age. Peri-menopausal and has been on hormonal replacement therapy, diagnosed with rectal cancer (low rectal

mass rT3, N0, M0) two years ago and completed treatment about 18 months ago (treatment offered: low anterior resection with ostomy). Generally feeling well, but finds it difficult to cope with urinary urgency and incontinence. Recent follow-up investigations: Pap smear normal; thyroid function tests normal; liver function tests normal; Vitamin D level normal; recent carcinoembryonic antigen test 3 ng/ml; CT scan of abdomen normal and no evidence of urinary tract infection. On examination no jaundice, anaemia, cyanosis, oedema, lymphadenopathy, no abdominal tenderness, small uterus with no mass or tenderness; no vaginal bleeding and mucosa appears normal; no punch tenderness over the kidneys, however, the pelvic floor is weak.<br>Diagnosis: Urinary dysfunction secondary to LAR/radiation
**Case 4:** Doreen, 54 years of age. Over the past two months, she has been feeling nauseous and sick, with lower back pain (awakening in pain at night) and weight loss that concerns her. She was diagnosed with sigmoid adenocarcinoma (T2, N1, M0) 2.5 years ago and completed treatment two years ago (laparoscopic anterior resection and neo adjuvant therapy). Six months ago her carcinoembryonic antigen levels were 11 ng/ml; CT scan of pelvis/abdomen, small area of low attenuation near the left lateral margin of the suture line? With fluid collection. Positron emission tomography showed no evidence of distant metastatic disease; CXR clear; Pap smear and liver function tests normal. On examination, there is mild lower abdominal distension; tenderness of the lower abdomen (diffuse non-specific); bowel sounds +++; per rectal examination, red blood and stool on glove.<br>Diagnosis: Tumour recurrence
**Case 5:** Joan, 68 years of age, retired nurse. She has non-insulin-dependent diabetes mellitus but her blood sugar is under control. She has been on metformin 500 mg twice daily for her diabetes for many years. She can no longer take her dog for a walk. She is easily exhausted. She completed treatment for colon cancer (caecal cancer T3, N0, M0) a year ago but has been feeling tired most of the time. Treatment offered: laparoscopic right hemicolectomy; adjuvant chemotherapy—fluorouracil + leucovorin for six months. Recent carcinoembryonic antigen levels normal; recent HbA1c 5.4–5.6%; Pap smear normal; thyroid function tests normal; liver function tests normal; vitamin D level normal; mammogram normal; ophthalmologist

review normal. On examination, there is good eye contact; and emotional response is congruent.<br>Diagnosis: Chemotherapy-induced fatigue
**Case 6:** Kerry, 77 years, retired. She is asthmatic but her asthma is under control. She was treated for upper rectal cancer (T3, N1, M0) a year ago. Her bowels haven't settled since she completed the treatment and she has been experiencing diarrhoea, which has significantly affected her social life. Treatment offered: pre operative chemo and radiotherapy 5/52. Post-operative adjuvant chemotherapy. Recent carcinoembryonic antigen 3 ng/ml and CT scan abdomen normal. Pap smear normal; thyroid function, liver function, vitamin B levels, full blood count and urea/electrolyte/creatinine levels are all normal. On examination, there no evidence of jaundice, anaemia, cyanosis, oedema, lymphadenopathy, or dehydration. Per rectal examination reveals watery stool and no blood.<br>Diagnosis: Chronic radiation proctitis
