# Supplementary material for: Do Patients Treated for Colorectal Cancer Benefit from General Practitioner Support? A Video Vignette Study
Source: J Med Internet Res. 2015 Nov 5;17(11):e249. doi: 10.2196/jmir.4942 (PMC4642383; doi:10.2196/jmir.4942)
Supplement: Multimedia Appendix 3 [file jmir_v17i11e249_app3.pdf]

Appendix 1: Specific Recommendations for Management of Cases

Symptom	Action to be taken by GP
Case 1: Peripheral neuropathy	**Prescribe:** amitriptyline or low dose of carbamazepine<br>**Health advice:** inform patient there is potential that this may not improve or may improve slowly over time. If the fingers are still numb at 12–18 months post chemotherapy it is likely that this will be permanent.<br>**Refer:** to oncologist for consideration of pregabalin.
Case 2: Erectile dysfunction secondary to LAR	**Prescribe:** phosphodiesterase inhibitors are recommended as first-line therapy.<br>**Refer:** to the hospital for further management, such as second-line therapy including penile self-injectable drugs, intraurethral alprostadil or vacuum devices.<br>**Link:** patient to support services.
Case 3: Urinary dysfunction secondary to LAR/radiation	**Order tests:** to rule out cardiovascular causes.<br>**Refer:** to physiotherapist for physical exercise.<br>**Refer:** to the specialist for further neurological examination.
Case 4: Tumour recurrence	**Order tests:** CT, magnetic resonance imaging (MRI), positron emission tomography (PET) scans and endorectal ultrasound (1). Repeat PET scan, full blood count (FBC) and blood film, LFT, UEC.<br>**Refer:** back to the oncologist for further management.
Case 5: Fatigue	**Order tests:** assess causes, as management of cancer-related fatigue involves specific treatment for potentially reversible causes (i.e., treating anaemia, metabolic or endocrine abnormalities, as well as managing pain, insomnia, depression, or anxiety). Symptomatic measures when no obvious aetiology or reversible cause can be identified.<br>**Prescribe:** psychostimulants. For patients with severe fatigue in whom non-pharmacologic methods do not resolve fatigue, and anaemia and other medical conditions and symptoms causing fatigue are controlled, a therapeutic trial of a psychostimulant (methylphenidate,

	dexmethylphenidate or modafinil) is a reasonable option.
Case 6: Chronic radiation proctitis	**Order tests:** visual inspection of lower bowel by proctoscopy and/or sigmoidoscopy and/or colonoscopy, assess for anaemia, attend FBC and stool culture to rule out comorbidities.<br>**Prescribe:** if true diarrhoea is established, an antidiarrheal agent is recommended (often combined with stool bulking).<br>**Health advice:** anorectal dysfunction has its origins in nerve and muscle fibrosis, and may be ameliorated by pelvic floor exercises and bowel re-training.<br>**Refer:** if (significant) bleeding develops or is confirmed refer patient for endoscopic therapies, such as thermal coagulation, or surgical therapies, such as protectomy or diversion colostomy if the condition worsens.
